# A Synthetic Derivative of Antimicrobial Peptide Holothuroidin 2 from Mediterranean Sea Cucumber (*Holothuria tubulosa*) in the Control of *Listeria monocytogenes*

**DOI:** 10.3390/md17030159

**Published:** 2019-03-08

**Authors:** Maria Grazia Cusimano, Angelo Spinello, Giampaolo Barone, Domenico Schillaci, Stella Cascioferro, Alessandra Magistrato, Barbara Parrino, Vincenzo Arizza, Maria Vitale

**Affiliations:** 1 Dipartimento di Scienze Biologiche, Chimiche e Farmaceutiche, Università di Palermo, Via Archirafi 32, 90123 Palermo, Italy; mariagrazia.cusimano@unipa.it (M.G.C.); stellamaria.cascioferro@unipa.it (S.C.); barbara.parrino@unipa.it (B.P.); vincenzo.arizza@unipa.it (V.A.); 2 CNR-IOM-Democritos c/o International School for Advanced Studies (SISSA), Via Bonomea 265, 34136 Trieste, Italy; angelo.spinello@sissa.it (A.S.); alessandra.magistrato@sissa.it (A.M.); 3 Istituto Zooprofilattico della Sicilia, Via Gino Marinuzzi, 3, 90129 Palermo, Italy; marvitus@yahoo.com

**Keywords:** antimicrobial peptides, *Holothuria tubulosa*, foodborne pathogens, *Listeria monocytogenes*, biofilm

## Abstract

Due to the limited number of available antibiotics, antimicrobial peptides (AMPs) are considered antimicrobial candidates to fight difficult-to-treat infections such as those associated with biofilms. Marine environments are precious sources of AMPs, as shown by the recent discovery of antibiofilm properties of Holothuroidin 2 (H2), an AMP produced by the Mediterranean sea cucumber *Holothuria tubulosa.* In this study, we considered the properties of a new H2 derivative, named H2d, and we tested it against seven strains of the dangerous foodborne pathogen *Listeria monocytogenes*. This peptide was more active than H2 in inhibiting the growth of planktonic *L. monocytogenes* and was able to interfere with biofilm formation at sub-minimum inhibitory concentrations (MICs). Atomic-level molecular dynamics (MD) simulations revealed insights related to the enhanced inhibitory activity of H2d, showing that the peptide is characterized by a more defined tertiary structure with respect to its ancestor. This allows the peptide to better exhibit an amphipathic character, which is an essential requirement for the interaction with cell membranes, similarly to other AMPs. Altogether, these results support the potential use of our synthetic peptide, H2d, as a template for the development of novel AMP-based drugs able to fight foodborne that are resistant to conventional antibiotics.

## 1. Introduction

There is an urgent need for novel classes of antibiotics able to escape the most common resistance mechanisms of pathogens [1]. Antimicrobial peptides (AMPs) are currently considered ideal candidates for the development of new classes of antimicrobials effective against resistant pathogens. Their mechanism of action, which involves the perturbation of the membrane structure rather than the inhibition of specific cellular targets, makes them less sensitive to bacterial antibiotic resistance mechanisms [2]. Additionally, AMPs have also proven to be active against bacterial biofilms, surface-attached microbial communities able to adhere to biotic or abiotic surfaces. In biofilms, the bacteria are embedded in a matrix of a self-synthesized extracellular polymeric substance (EPS), which makes antibiotic entry difficult. Biofilm formation is considered a significant virulence factor for the majority of human pathogens [3].

In the struggle against antibiotic resistance, the development of efficacious antibiofilm therapies represents a goal of growing interest for the scientific community [4,5,6]. In fact, microbial cells in biofilms are thousand-folds more resistant to antibiotic therapies than the planktonic forms of the same bacterial strains, and biofilm-associated infections are a health issue of great concern [7]. Marine environments are important sources of AMPs [8]. Recently, when searching for new AMPs, we focused on coelomocytes (immune mediator cells) of Mediterranean echinoderms [9,10]. In particular, in the sea cucumber *Holothuria tubulosa* (Echinodermata), we identified two peptides, Holothuroidin 1 (H1) and Holothuroidin 2 (H2), which showed antimicrobial activity against free living and complex sessile community (biofilm) forms of a reference group of Gram-negative and Gram-positive human pathogens [10]. H1 and H2 are cationic peptides, 12 and 14 amino acids long, respectively, which have the same amino acidic sequence, except for the presence of two additional residues (alanine and serine) at the N-terminus of H2. The synthetic peptide H2 showed broad-spectrum activity at 12.5 mg/mL against all tested Gram-positive and Gram-negative strains. Both peptides were effective in inhibiting biofilm formation at a concentration of 3.1 mg/mL against staphylococcal and *Pseudomonas aeruginosa* strains. A comparison of the primary sequences highlighted that H1 shows a similarity of 42.8% with Pronectin, an AMP produced by a wasp (*Agelaia papilles*), and that H2 shows a similarity of 39.1% with Frenatin 3, an AMP isolated in the skin secretions from the Australian frog *Litoria infrafrenata* [11].

To obtain more potent AMPs, a new H2 derivative (H2d) was designed and tested against strains of the relevant foodborne pathogen *Listeria monocytogenes*, which is responsible for serious infection (listeriosis) in humans. Despite listeriosis being an uncommon disease compared to other foodborne illnesses, it is potentially fatal with a mortality rate of 20–30%. Additionally, an outbreak of listeriosis has a significant economic impact causing not only a burden related to healthcare and treatment, but also economic losses in food industry businesses [12]. This microorganism is also capable of adhering to, and forming biofilms on, surfaces such as metal, plastic, glass, stainless steel, or rubber. For this reason, growth control of this microorganism as a sessile community is a pursued goal [13]. Moreover, it is also responsible for contamination in the food industry because of its environmental persistence. *L. monocytogenes* strains can persist for years or decades in the food industry. Recent research focused on the role of biofilm formation on, and tolerance to, sanitation standard operating procedures, that is, daily cleaning by rinsing, soaping, etc. [14]. Hence, new agents able to counteract its growth in both planktonic and biofilm form are strongly required [15,16].

Here, we describe the in vitro anti-*Listeria* activity of the three peptides, H2, H2d, and the amino acid fragment added to H2 to obtain H2d (Tag), against planktonic and biofilm growth of four reference strains of *L. monocytogenes* ATCC 19114, ATCC 19115, ATCC 7644, and NCTC 18890, and three isolates from food of *L. monocytogenes*, LM89, LM101, and LM103.

## 2. Results

The three chemically synthesized peptides used in this study were purchased from a biotechnology company. H2 was synthesized according to the sequence reported in our previous study [11], and H2d was designed by adding, at the N-terminus of H2 a histidine-rich head, 22 amino acids long. This fragment (named Tag) shows a similarity of 38.4% with Histatin 4, an AMP found in human saliva, which showed antimicrobial and antifungal properties [17]. Finally, the peptide Tag was also synthesized. The sequences of the three peptides are reported in Table 1.

Physicochemical properties of AMPs are relevant to estimate their antibacterial potency and potential mechanism of action. The most significant parameters of H2, Tag, and H2d (Table 2) were analyzed using the Antimicrobial Peptide Database [18] and Half-Life of Peptides (HLP), a useful tool to predict the half-life of peptides in a biological proteolytic environment [19].

The in silico analysis revealed important physicochemical properties of the tested peptides, giving insight into their potential mechanism of action. As an example, the Boman index showed that Tag has a higher affinity for proteins than for lipid bilayers, because a more hydrophilic peptide tends to have a more positive index. Since the antimicrobial activity highly depends on the peptide tertiary structure, the three peptides were generated using the PEP-FOLD3 algorithm [20] in order to shed light on the mechanism of action. This program [21] performs a de novo prediction of the tertiary structure from the amino acid primary sequence. However, it is important to remark that de novo prediction currently remains a challenging task [22]. The best models obtained for each peptide, along with the second and third proposed structures, in terms of their energetic stability, are reported in Supplementary Figure S1. Interestingly, while the shortest peptide, H2, is always predicted as a α-helix and the other proposed models are almost identical, slight differences appear for the other two peptides, with Tag being more unstructured.

### 2.1. Molecular Dynamics Simulations

**Figure 1:**
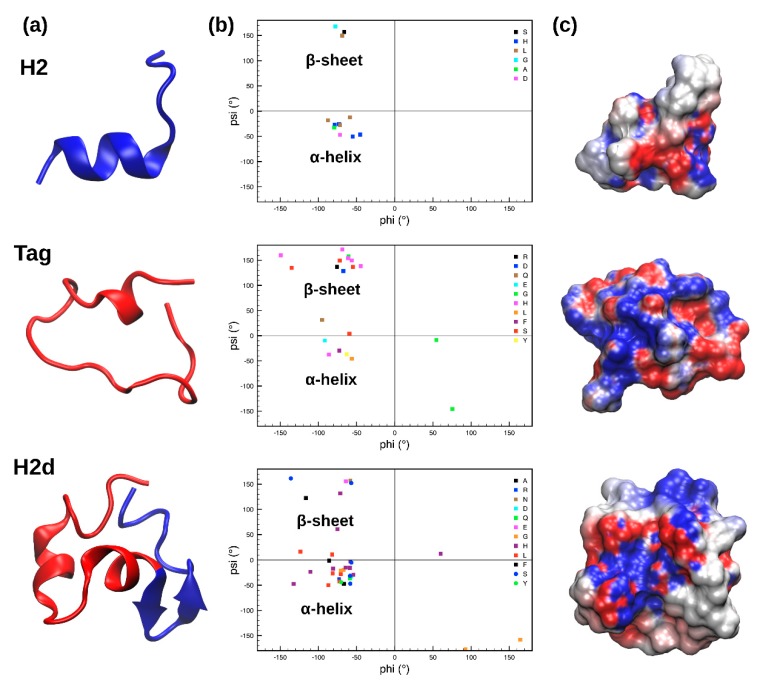
(**a**) Structure of the most representative cluster of H2, Tag, and H2d obtained from the molecular dynamics (MD) simulations; blue and red colors respectively identify the H2 and Tag moieties in H2d. (**b**) Ramachandran plots showing the values of the psi and phi angles assumed by each residue. (**c**) Electrostatic potential of the peptides structures, shown as surfaces; red and blue colors are used for negative and positive values, respectively.

To assess the stability of the lowest energy models obtained by PEP-FOLD3 and to relax their structure in a physiological environment, 1 μs MD simulations were performed on each of the three peptides. The most representative structures were extracted for the MD trajectories by using a cluster analysis (Figure 1a). An analysis of their secondary structure, as evidenced by the Ramachandran plots for the most representative clusters (Figure 1b), reveals that H2 and H2d have a higher number of residues adopting psi and phi angles compatible with a α-helical motif, while Tag possesses a higher proportion of residues in a β-sheet conformation.

During the MD simulation, the shorter peptides, H2 and Tag, are very flexible (as shown by the root mean square deviation (RMSD) plot reported in Supplementary Figure S2) and capable of exploring a wide portion of the conformational space, providing more distinct secondary structures. In particular, H2 partially retains the originally predicted α-helical conformation, spanning from His6 to Leu13 (Figure 1a). The first cluster of Tag is more unstructured, except for small α-helical content in the region between Asn16 and Gln20. The remaining amino acids adopt a linear conformation, with psi and phi angles typical of a β-sheet. Interestingly, when H2 and Tag are merged together to form H2d, the resulting peptide is less flexible (Supplementary Figure S2) but still adopts different secondary structural motifs in comparison with the ones observed for H2 and Tag. In particular, the Tag moiety of H2d (H2d red portion in Figure 1a) assumes an α-helical fold, while the H2 moiety (H2d blue part in Figure 1a) switches from an α-helix to a β-sheet in the region between Ala23 and His29.

Finally, the electrostatic potential calculated for the three peptides, as shown in Figure 1c, reveals that H2d has a more dipolar or amphipathic nature, compared to the two other investigated peptides, displaying a clear separation between charged (red and blue for negative and positive values, respectively) and uncharged regions (white surface).

### 2.2. Anti-Listeria Activity In Vitro against Planktonic and Biofilm Growth

The three peptides were tested in vitro against four reference strains of *L. monocytogenes*, ATCC 19114, ATCC 19115, ATCC 7644, and NCTC 18890, and three food isolates of *L. monocytogenes*, LM89, LM101, and LM103. H2d showed better activity compared to H2, which was inactive against all tested strains at the maximum tested concentration of 5 mg/mL, with the exception of the reference strain *L. monocytogenes* ATCC19115. The peptide Tag showed no activity against the tested strains at screening concentration of 5 mg/mL. Two different susceptibility culture media were used for comparative purposes, and the activities are summarized in Table 3 and Table 4.

The ability of the tested *L. monocytogenes* strains to grow as a biofilm, in vitro, was evaluated by determining optical density (OD) of adhered biomass at 600 nm; this revealed a range from a higher OD value of 0.888 for strain LM101 isolated from food, to a lower value of 0.251 for reference strain *L. monocytogenes* NCTC 18890.

**Figure 2:**
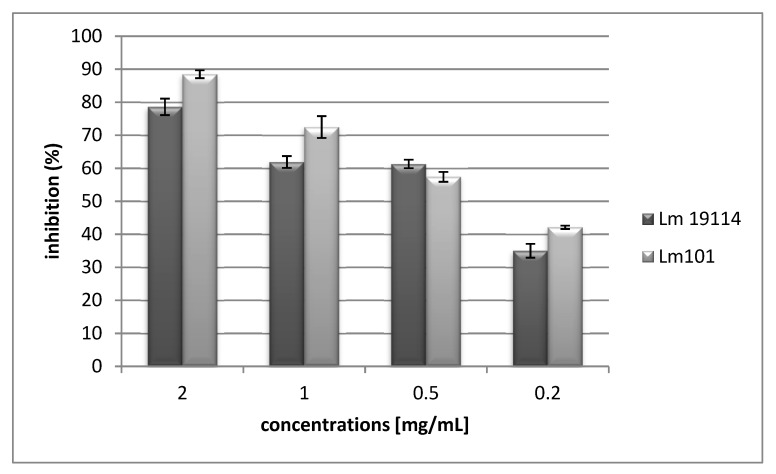
Inhibition of biofilm formation.

The peptide H2d was tested for its ability to interfere with biofilm formation of the best biofilm producers among isolates and reference strains: *L. monocytogenes* LM 101 and *L. monocytogenes* ATCC19114. The results in terms of inhibition of biofilm formation are presented in Figure 2.

H2d at a sub-MIC value of 2 mg/mL (0.49 mM), proved to effectively inhibit biofilm formation of *L. monocytogenes* 101 and *L. monocytogenes* ATCC19114 with a percentage of inhibition value respectively equal to 88.5% and 78.6% (Figure 2). The values of biofilm inhibition concentration (BIC_50_), in interfering with biofilm formation, were 0.31 mg/mL (0.076 mM) against *L. monocytogenes* LM101, and 0.47 mg/mL (0.11mM) towards *L. monocytogenes* ATCC 19114.

## 3. Discussion

Most of the clinical and environmental *Listeria* spp. isolates are susceptible to antibiotics active against Gram-positive bacteria. However, there is growing evidence of multiresistant strains of *Listeria* spp. [23,24,25,26,27]. The emergence of drug-resistant bacteria has focused attention towards the development of antimicrobial peptides as novel anti-infective agents. Human defensins, cathelicidins, and AMPs from bacteria (bacteriocins), viruses, plants, vertebrates, and invertebrates have the potential to replace or supplement traditional antibiotics. *L. monocytogenes* can be isolated from the work surfaces of food-processing establishments even after routine cleaning and disinfection procedures, due to its ability to form a biofilm that is protective against environmental stress.

The H2d peptide is derived from the addition of a peptide, Tag, consisting of 22 amino acids (MRGSHHHHHHGSSGENLYFQSL), to H2 AMP isolated in *H. tubulosa* (ASHLGHHALDHLLK) [11], resulting in a larger peptide (MRGSHHHHHHGSSGENLYFQSLASHLGHHALDHLLK) of 36 amino acids. An almost identical amino acid sequence was used previously to build a synthetic peptide recombinant paracentrin 1 (RP1) using an AMP from *Paracentrotus lividus* Paracentrin 1 (P1) [28]. RP1 was considerably more active than P1 against the planktonic forms of *Staphylococcus aureus* ATCC 25923 and *P. aeruginosa* ATCC 15442 at concentrations of 50 µg/mL. Moreover, it was able to inhibit biofilm formation of staphylococcal and *P. aeruginosa* strains at concentrations equal to 5.0 µg/mL and 10.7 µg/mL, respectively. In line with our previous findings, MIC assays in this study showed that the synthetic peptide H2d is more effective than H2 in inhibiting *L. monocytogenes* strains.

Determination of the physicochemical properties of the active peptides showed a hydrophobic ratio of 27% for H2d. Peptides with higher hydrophobicity were shown to undergo a structural transition in contact with bacterial-like membranes, and to have a propensity to form helical or sheet structures, thereby triggering the mode of action of AMPs. The presence of hydrophilic residues in H2d may help in rationalizing its mode of action. Furthermore, in silico analysis based on the antimicrobial peptide database predicted the potentiality of H2d as an AMP. H2d shares some degree of sequence similarity with known AMPs in the literature, such as, Pep27 (33.3%), a bacteriocin from Gram-positive bacteria *Streptococcus pneumoniae* [29] and GHH20, a His-rich glycoprotein with antifungal activity against *Candida parapsilosis* and *Candidaalbicans* isolated from humans [30]. MD simulations suggest that H2d may assume a more defined tertiary structure in comparison with the more flexible H2 and Tag peptides. Furthermore, although the three peptides have a neutral total charge, the analysis of the electrostatic potential (Figure 1c) clearly shows a more pronounced amphipathic nature of H2d [31]. This property, usually shared among many AMPs, is essential for their interaction with bacterial membranes and it can be further enhanced upon interaction, since the hydrophilic fraction of the peptide will interact with the phospholipid heads of the membrane, while the hydrophobic part will remain buried in the lipid double layer [32]. These findings are in line with the higher inhibitory power displayed by H2d against the strains of *L. monocytogenes* investigated.

The antimicrobial activity of H2d is effective not only on planktonic cells but also against the biofilm formation of *L. monocytogenes.* Numerous studies have shown that these bacteria are capable of adhering to, and forming biofilm on, metal, glass, or rubber surfaces [33,34,35,36,37,38]. Biofilm formed in food processing environments is particularly important as it can act as a source of chronic and persistent microbial contamination that may lead to food spoilage or transmission of diseases. Bacteria in biofilms exhibit enhanced resistance to cleaning and sanitation [39,40]. In recent years, numerous reviews and articles have been published demonstrating how AMPs extracted from invertebrates can control the growth of *L. monocytogenes*, providing detailed information on the physicochemical properties, activity, and mechanism of action [41,42].

Our results support the potential use of H2d in the struggle against such important foodborne pathogens resistant to conventional antimicrobials, in particular when they grow as a biofilm responsible for environmental persistence and multifactorial resistance to antimicrobials.

## 4. Materials and Methods

### 4.1. In Silico Analysis

The “APD3: Antimicrobial Peptide Calculator and Predictor” tool of the Antimicrobial Peptide Database (APD) [18] was used to identify the following general characteristics of antimicrobial peptides, such as hydrophobicity, affinity to attach to other proteins (Boman index) and similarities with known AMPs. Also, the “HLP: Web server for predicting half-life of peptides in intestine-like environment” allowed the half-life and stability of the new peptides in an intestine-like, proteolytic environment to be theorized upon [19].

### 4.2. Molecular Dynamics Simulations

The PEP-FOLD3 webserver [20] was used in order to obtain a folded structure of H2, Tag, and H2d peptides. For each structure, 100 runs were performed, and clusters were sorted by sOPEP energy. This software, used for the de novo prediction of peptide and small protein tertiary structures, first predicts a limited number of local conformations using a hidden Markov model-derived structural alphabet. Afterwards, it assembles them through a greedy procedure using a coarse-grained energy function [43]. The lowest energy model was taken as a starting point for subsequent simulations, as seen in Figure 1a. In particular, the stability of the peptide conformations in physiological conditions was assessed by MD simulations, following reported procedures [44,45]. MD simulations were performed using a Particle Mesh Ewald MD (PMEMD) module of Amber18 [46], using the Amber99SB-ILDN force field [47]. The three peptides were solvated in a truncated octahedron box with a distance between the walls and the solute atoms of 12 Å, and filled with TIP3P water molecules [48]. A cutoff of 10 Å for the non-bonded interactions and a time step of 2 fs was used. The final systems were heated for 1 ns to achieve a final temperature of 300 K using a Langevin thermostat [49]. The box size was equilibrated for 1 ns in the NPT (isothermal-isobaric) ensemble. The pression control (1 atm) was performed using a Berendsen barostat [50]. Finally, production NVT (canonical ensemble) runs of 1 μs were performed for each peptide, for a cumulative simulation time of 3 μs. Figures and plots were obtained by the VMD software (1.9.2, University of Illinois, Urbana-Champaign, IL, USA) [51]. Clustering analysis was performed using a hierarchical agglomerative approach via the AmberTools ptraj module [46]. Electrostatic potential was calculated using PDB2PQR server (2.1.1, Pacific Northwest National Laboratory, Richland, WA, USA) [52].

### 4.3. Peptides Synthesis

The tested peptides were synthesized by GenScript Biotech, Piscataway Township, NJ, USA, according to our design and indications. Fluorenylmethyloxycarbonyl protecting group (Fmoc) solid phase technology was used to obtain the peptides. The purity (95%) of each peptide was determined by high performance liquid chromatography (HPLC) and mass spectrometry (MS) analyses.

### 4.4. Minimum Inhibitory Concentration Determination

Minimum inhibitory concentrations (MICs) of the synthesized peptides from free living forms (planktonic) were determined using a reference micromethod [53]. The two most common conventional susceptibility testing media, Mueller–Hinton Broth (MHB) and Mueller–Hinton Broth cation adjusted (MHB2), were employed for comparative purposes. The analyses were performed on four reference strains of *L. monocytogenes*, ATCC 19114, ATCC 19115, ATCC 7644, and NCTC 18890, and three isolates from food *L. monocytogenes*, LM 89, LM 103, and LM 104. Positive growth controls without tested samples and negative controls without bacteria were included. Each assay was performed in triplicates and repeated at least twice.

### 4.5. Evaluation of Biofilm Formation

Test tubes with 5 mL tryptic soy broth (TSB) containing 2% (w/v) glucose were inoculated with a loopful of a 24 h culture from tryptic soy agr (TSA) tubes and incubated at 37 °C for 24 h. After incubation, the required number of wells of a sterile polystyrene flat-bottom 96-well plate (JET BIOFIL^®^, Guangzhou Jet Bio-Filtration Co., Ltd, Guangzhou, China) were loaded with 200 μL of the medium (TSB with 2% glucose) and 2.5 μL of bacterial suspension at an OD at 570 nm of about 0.040, corresponding to a viable count of ~10^6^ cfu/mL. The microtiter plate was sealed with parafilm and incubated at 37 °C for 24 h. Wells were washed twice with sterile 1× PBS and stained with 100 μL of 0.1% crystal violet solution for 10 min at room temperature. Surplus solution was discharged and the plate was washed twice using tap water. A volume of 200 μL of ethanol was added into each stained well to solubilize dye, for 10 min at room temperature. OD was read at a wavelength of 540 nm using a plate reader (Glomax Multidetection System TM297 Promega, Milano, Italy). The experiments were performed at least in triplicates, and three independent experiments were run.

### 4.6. Inhibition of Biofilm Formation (Crystal Violet Method)

Bacterial strains were incubated in test tubes with 5 mL TSB containing 2% (w/v) glucose at 37 °C for 24 h. After that, the bacterial suspensions were diluted and 2.5 μL of the diluted suspension was placed into each well of a sterile flat-bottom 96-well, as described above (Evaluation of Biofilm Formation). Aliquots at sub-MIC concentration of peptide H2d were directly added to the wells to obtain concentrations ranging from 2to 0.2 mg/mL. Plates were incubated at 37 °C for 24 h. After biofilm growth, wells were washed and stained with crystal violet solution as previously described. Surplus solution was discharged, and the plate was washed as earlier described. Each stained well was processed with ethanol as described above. OD was read with wavelength of 540 nm using a microplate reader (Glomax Multidetection System Promega, Milano, Italy). BIC_50_, that is, the concentration at which the percentage of inhibition of biofilm formation (see below) is equal to 50%, was obtained by comparing the ODs of control wells with that of the sample wells, and the value was calculated using a linear regression graph in Excel. The experiments were performed at least in triplicates, and three independent experiments were run.

The percentage of inhibition was calculated by using the following the formula:

% of inhibition = (OD growth control − OD sample)/OD growth control) × 100

## Figures and Tables

**Table 1:** Primary sequences of Holothuroidin 2 (H2), H2 derivative (H2d), and the amino acid fragment added to H2 to obtain H2d (Tag).

Peptide	Sequence
H2	ASHLGHHALDHLLK
Tag	MRGSHHHHHHGSSGENLYFQSL
H2d	MRGSHHHHHHGSSGENLYFQSLASHLGHHALDHLLK

**Table 2:** Principal physicochemical parameters of the three peptides.

Chemical Physical Properties	Peptides		
	H2	Tag	H2d
Theoretical mass (Da)	1548.7	2555.7	4086.5
Isoelectric point	6.68	6.37	6.41
Hydrophobic ratio	42%	18%	27%
Protein-binding potential Boman index (kcal/mol)	0.86	2.61	1.93
Half-life (s)	1.13	0.73	0.64

**Table 3:** Anti-*Listeria* activity in Mueller–Hinton Broth (MHB) medium.

Minimum Inhibitory Concentration (MIC)mg/mL and [mM]							
Peptides	*L. monocytogenes* Strains						
	ATCC19114	ATCC19115	ATCC7644	NCTC18890	89	101	103
**H2**	>5 [3.2]	5 [3.2]	>5 [3.2]	>5 [3.2]	>5 [3.2]	>5 [3.2]	>5 [3.2]
**Tag**	>5 [2.0]	>5 [2.0]	>5 [2.0]	>5 [2.0]	>5 [2.0]	>5 [2.0]	>5 [2.0]
**H2d**	2.5 [0.6]	2.5 [0.6]	2.5 [0.6]	5 [1.2]	1.2 [0.3]	2.5 [0.6]	2.5 [0.6]

**Table 4:** Anti-*Listeria* activity in Mueller–Hinton Broth cation adjusted (MHB2) medium.

	MIC mg/mL and [mM]						
Peptides	*L. monocytogenes* Strains						
	ATCC19114	ATCC19115	ATCC7644	NCTC18890	89	101	103
**H2**	>5 [3.2]	5 [3.2]	>5 [3.2]	>5 [3.2]	>5 [3.2]	>5 [3.2]	>5 [3.2]
**Tag**	>5 [2.0]	>5 [2.0]	>5 [2.0]	>5 [2.0]	>5 [2.0]	>5 [2.0]	>5 [2.0]
**H2d**	2.5 [0.6]	1.2 [0.3]	2.5 [0.6]	5 [1.2]	2.5 [0.6]	2.5 [0.6]	2.5 [0.6]

## Supplementary Materials

The following are available online at https://www.mdpi.com/1660-3397/17/3/159/s1, Figure S1: Structures of the best three models predicted by PEP-FOLD3; Figure S2: Root mean square deviation (RMSD) of the MD simulations.
