# Supplementary material for: A Synthetic Derivative of Antimicrobial Peptide Holothuroidin 2 from Mediterranean Sea Cucumber (Holothuria tubulosa) in the Control of Listeria monocytogenes
Source: Mar Drugs. 2019 Mar 8;17(3):159. doi: 10.3390/md17030159 (PMC6471310; doi:10.3390/md17030159)
Supplement: Supplementary file 1 [file marinedrugs-17-00159-s001.pdf]

marine drugs

Supplementary Materials

A Synthetic Derivative of Antimicrobial Peptide Holothuroidin 2 from Mediterranean Sea Cucumber (*Holothuria tubulosa*) in the Control of *Listeria monocytogenes*

Maria Grazia Cusimano [1,†], Angelo Spinello [2,†], Giampaolo Barone [1,*], Domenico Schillaci [1,*], Stella Cascioferro [1], Alessandra Magistrato [2], Barbara Parrino [1], Vincenzo Arizza [1] and Maria Vitale [3]

1 Dipartimento di Scienze Biologiche, Chimiche e Farmaceutiche, Università di Palermo, Via Archirafi 32, 90123, Palermo, Italy; mariagrazia.cusimano@unipa.it (M.G.C.); stellamaria.cascioferro@unipa.it (S.C.); barbara.parrino@unipa.it (B.P.); vincenzo.arizza@unipa.it (V.A.)
2 CNR-IOM-Democritos c/o International School for Advanced Studies (SISSA), Via Bonomea 265, 34136, Trieste, Italy; angelo.spinello@sissa.it (A.S.); alessandra.magistrato@sissa.it (A.M.)
3 Istituto Zooprofilattico della Sicilia, Via Gino Marinuzzi, 3, 90129 Palermo, Italy; marvitus@yahoo.com
* Correspondence: giampaolo.barone@unipa.it (G.B.); domenico.schillaci@unipa.it (D.S); Tel.: +39-09123897973 (G.B.); +39-09123891914 (D.S.)
† These authors contributed equally to this work.

Content

Figure S1 Page 2

Figure S2 Page 3

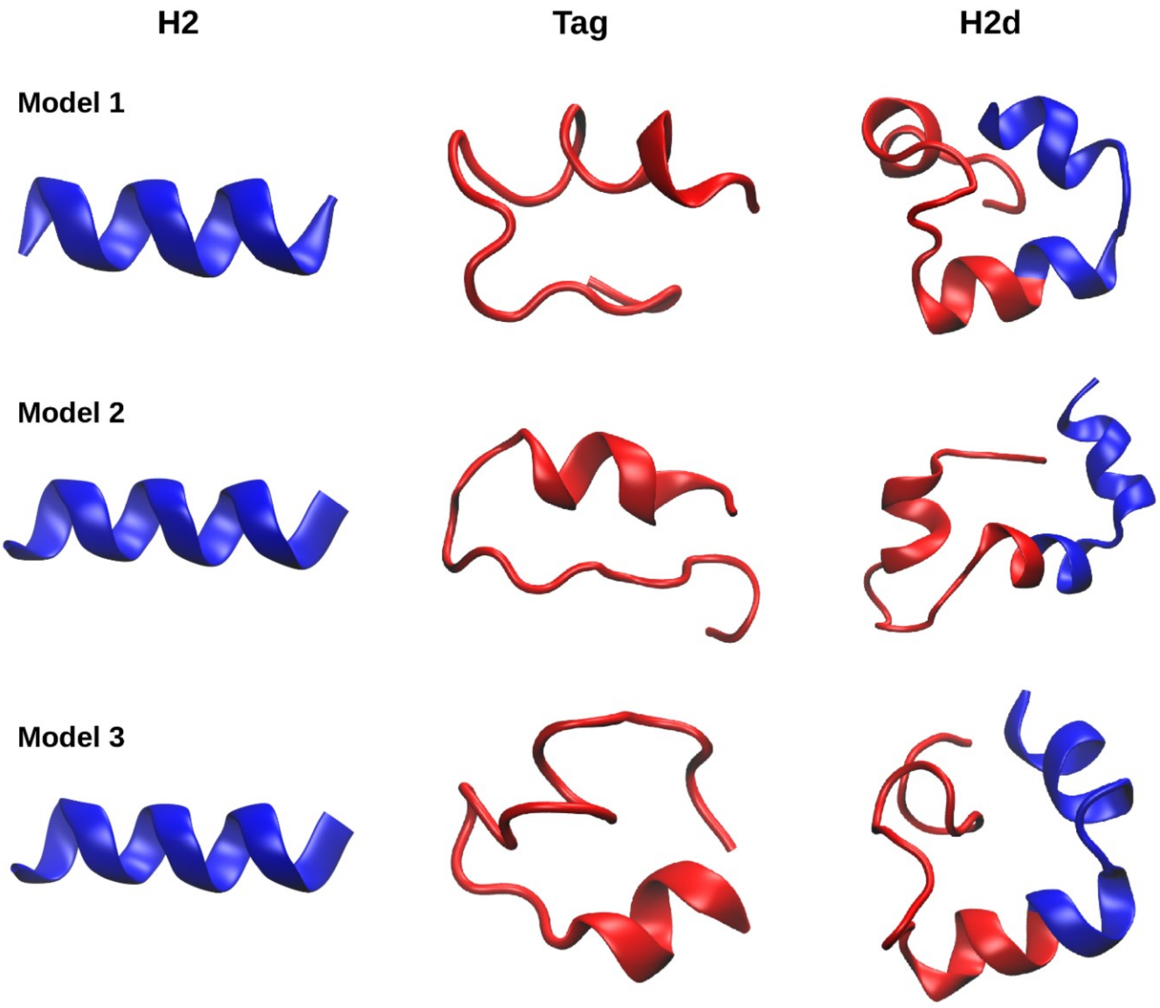

Figure S1. Structures of the best three models predicted by PEP-FOLD3 software for H2, Tag and H2d peptides.

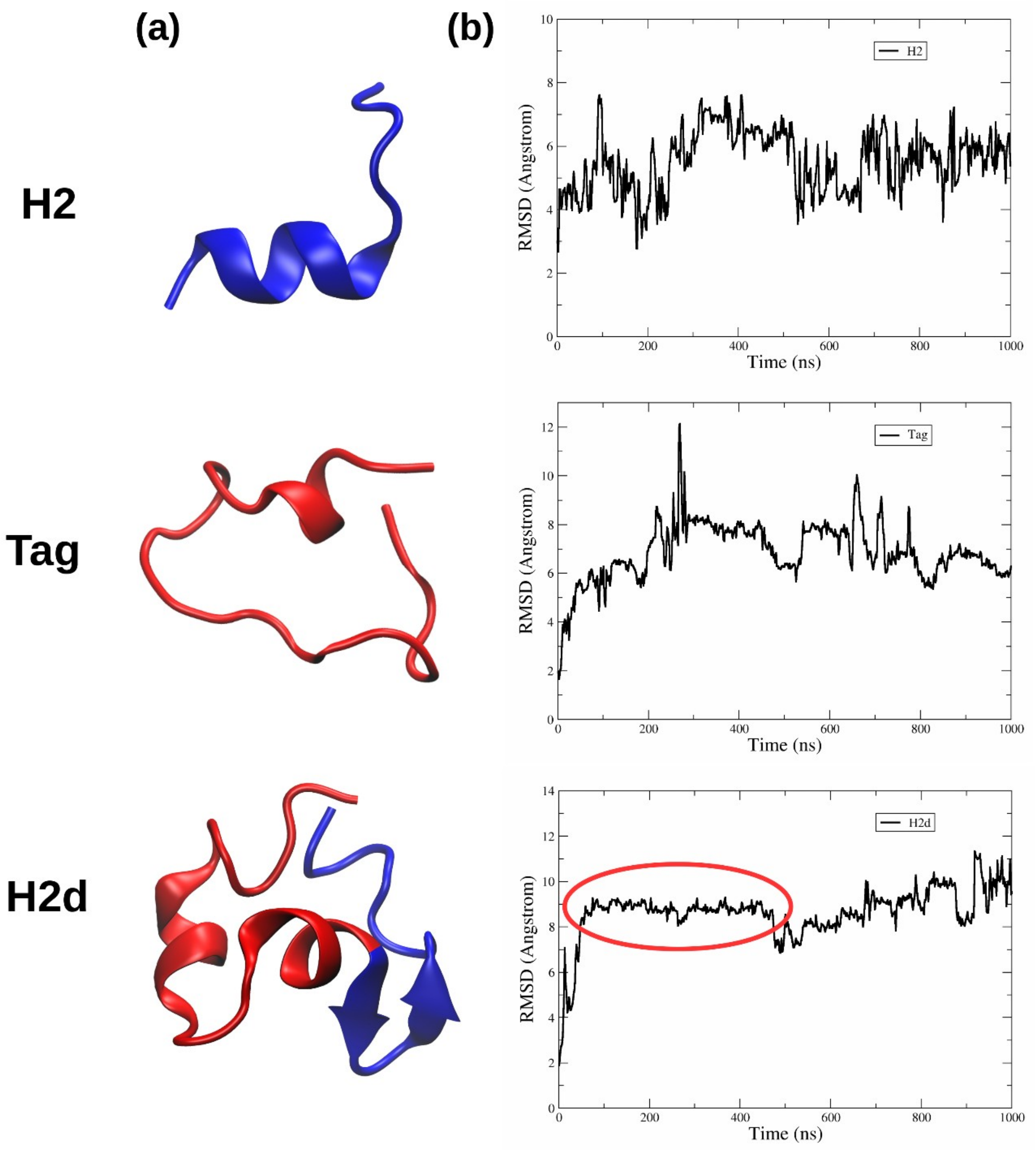

Figure S2. (**a**) The most representative cluster extracted from the Molecular Dynamics (MD) trajectories. (**b**) Root Mean Square Deviation (RMSD) of the MD simulations. H2 and Tag peptides are more flexible, while H2d adopts a stable tertiary structure for about 500 ns (highlighted in red).
